# Structures of Two New Flavonoids and Effects of Licorice Phenolics on Vancomycin-Resistant *Enterococcus* Species

**DOI:** 10.3390/molecules19043883

**Published:** 2014-03-28

**Authors:** Eerdunbayaer, Mohamed A. A. Orabi, Hiroe Aoyama, Teruo Kuroda, Tsutomu Hatano

**Affiliations:** 1 Department of Natural Product Chemistry, Okayama University, Graduate School of Medicine, Dentistry and Pharmaceutical Sciences, Tsushima-naka, Kita-ku, Okayama 700-8530, Japan; E-Mails: eerdun3@163.com (E.); m_a_orabi@yahoo.com (M.A.A.O.); ph20101@s.okayama-u.ac.jp (H.A.); 2 Faculty of Pharmacy, Al-Azhar University, Assiut 71524, Egypt; 3 Drug Discovery Technology Center, Okayama University Graduate School of Medicine, Dentistry and Pharmaceutical Sciences, Tsushima-naka, Kita-ku, Okayama 700-8530, Japan; E-Mail: tkuroda@cc.okayama-u.ac.jp

**Keywords:** licorice, *Glycyrrhiza uralensis*, phenolics, flavonoid, 3-arylcoumarin, 2-arylcoumarone, VRE, antibacterial effect

## Abstract

Since our previous study revealed that several licorice phenolics have antibacterial effects on methicillin-resistant *Staphylococcus aureus* (MRSA), and suppressive effects on the oxacillin resistance of MRSA, we further investigated effectiveness of licorice constituents on vancomycin-resistant *Enterococcus* (VRE) bacteria, and purified 32 phenolic compounds. Two flavonoids among them were characterized structurally, and identified their structures as demethylglycyrol (**31**) and 5,7-di-*O*-methylluteone (**32**), respectively. Examination of antibacterial effects of licorice phenolics showed that 3-arylcoumarins such as licoarylcoumarin (**9**) and glycycoumarin (**26**), and 2-arylcoumarones such as gancaonin I (**17**), have moderate to potent antibacterial effects on the VRE strains used in this study.

## 1. Introduction

Licorice is one of the most frequently used natural drugs in Asian traditional medicines. It produces various types of phenolic constituents, in addition to glycyrrhizin and related triterpene glycosides. Recently, the biological activities of licorice extracts and ingredients have attracted many researchers, and their effects in the treatment for different human diseases such as cancer, atherosclerosis, gastric ulcers, hepatitis and immunodeficiency have been summarized in some reviews [1,2,3]. Potential beneficial effects of licorice in common oro-dental diseases were also discussed in a review article [4]. Potent antibacterial activities of licorice phenolics against bacterial strains such as *Helicobacter pylori*, cariogenic bacterial species, *Streptococcus mutans* and *Streptococcus sobrinus*, and periodontopathogenic species, *Porphyromonas gingivalis* and *Prevotella intermedia*, were also reported [5,6,7,8]. Our previous investigation revealed that several naturally occurring compounds showed potent antibacterial effects on methicillin-resistant *Staphylococcus aureus* (MRSA) [9,10,11], and some of the licorice phenolics, such as licoricidin (**1**), showed suppressing effects on the oxacillin resistance of MRSA [11].

Among drug-resistant bacteria vancomycin-resistant *Enterococci* (VRE) is a serious menace for patients in hospitals. Just a few drugs such as linezolid show bacteriostatic activity against vancomycin-resistant strains of E. faecium and E. faecalis, and a combination of quinupristin and dalfopristin, which have bactericidal activity against most drug-resistant *staphylococci*, *streptococci*, and *pneumococci*, appears to be bacteriostatic against E. faecium, and is not active against Enterococcus faecalis [12]. Therefore, we have investigated on the effective constituents of licorice (licorice based on *Glycyrrhiza uralensis*) on VRE, and found that some phenolics among them had potent to moderate antibacterial effects on VRE. Since two compounds among the phenolics isolated from licorice have not yet been characterized, their structures were established in the present study. This paper describes structural evidence of the two compounds and effects of licorice phenolics on VRE. Worthy that antimicrobial activities of extracts of leaves and roots of *Glycyrrhiza* species were previously studied [13,14] against several bacterial strains including *Enterococcus faecalis*. Although a paper reported gancaonin I (**17**) as a compound with the anti-VRE effect [15], our study revealed several pure phenolic compounds from licorice should also be considered as lead compound candidates for new anti-VRE drugs, as shown below.

## 2. Results and Discussion

**Figure 1:**
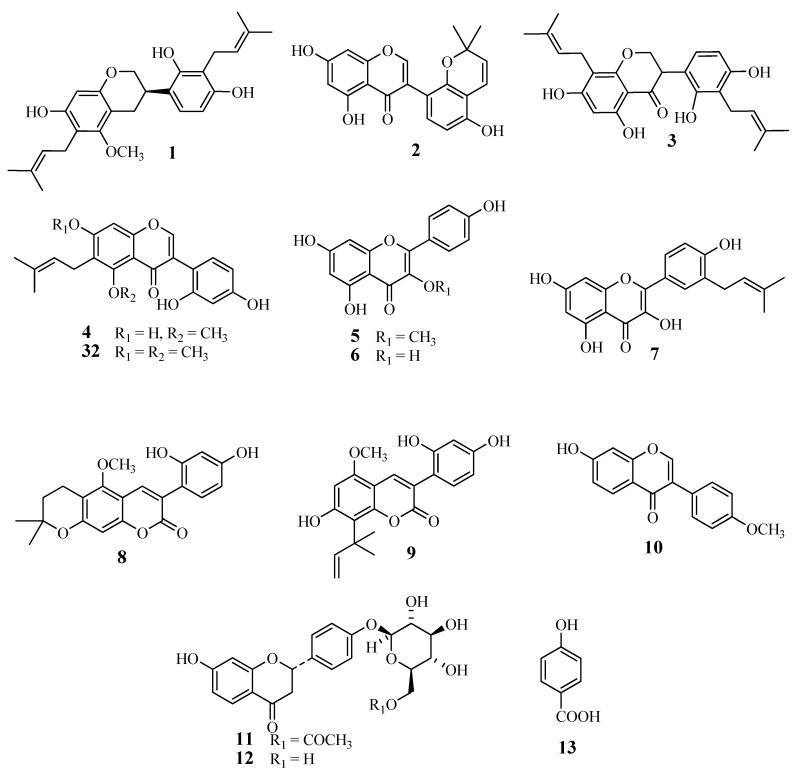
Chemical structures of compounds **1**‒**13** and **32** isolated from root and stolon of *Glycyrrhiza uralensis*.

**Figure 2:**
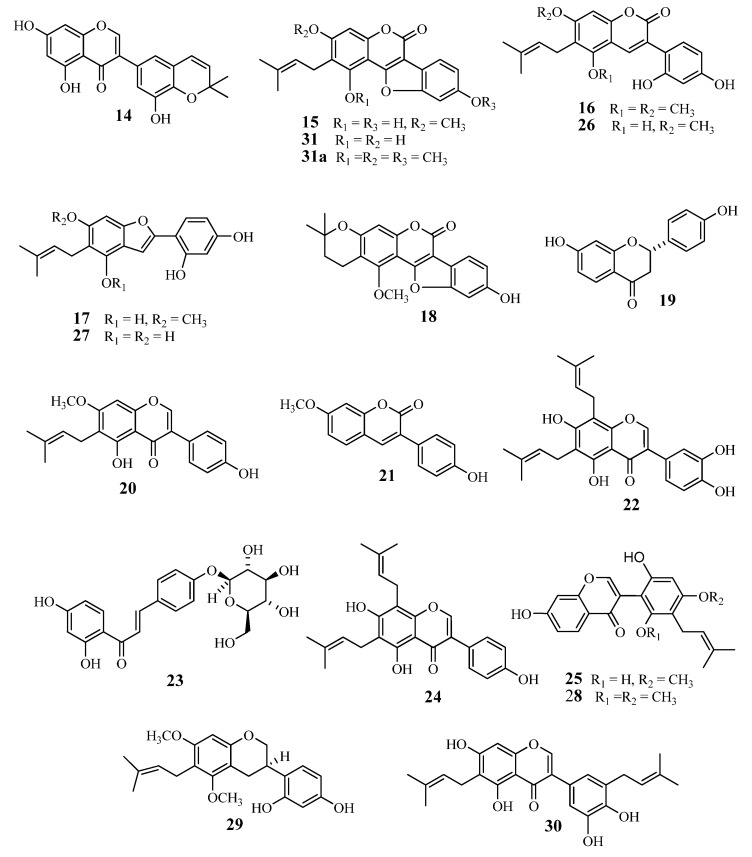
Chemical structures of compounds **14**‒**31** isolated from root and stolon of *Glycyrrhiza uralensis*.

The licorice phenolics (Figure 1 and Figure 2) were isolated from the ethyl acetate extract in the following way: the extract was subjected to countercurrent distribution with CHCl_3_–CH_3_OH–H_2_O, and the less polar fractions were respectively chromatographed on silica gel, ODS-gel, and MCI-gel CHP-20P, to give licoricidin (**1**) [16], allolicoisoflavone B (**2**) [17], 3'-(*γ*,*γ*-dimethylallyl)-kievitone (**3**) [18], 7-*O*-methylluteone (**4**) [19], kaempferol 3-*O*-methyl ether (**5**) [20], and kaempferol (**6**) [21], and fractions containing phenolics. Those fractions were purified by preparative TLC on silica gel or by preparative HPLC to give isolicoflavonol (**7**) [22], isoglycycoumarin (**8**) [23], licoarylcoumarin (**9**) [24], formononetin (**10**) [25], and 6"-*O*-acetylliquiritin (**11**) [26]. On the other hand, the remaining part of the ethyl acetate fraction was directly subjected to column chromatography on ODS-gel, and fractions from the column were further purified by column chromatography on MCI-gel CHP-20P, and by preparative HPLC or preparative TLC, to give liquiritin (**12**) [27], *p*-hydroxybenzoic acid (**13**), semilicoisoflavone B (**14**) [28], glycyrol (**15**) [29], glycyrin (**16**) [29], gancaonin I (**17**) [30], isoglycyrol (**18**) [31], liquiritigenin (**19**) [27], gancaonin G (**20**) [32], 3-(*p*-hydroxyphenyl)-7-methoxycoumarin (**21**) [33], 6,8-diprenylorobol (**22**) [34], isoliquiritin (**23**) [35], 8-(*γ*,*γ*-dimethylallyl)-wighteone (**24**) [36], glicoricone (**25**) [37], glycycoumarin (**26**) [38], licocoumarone (**27**) [29], licoricone (**28**) [39], glyasperin D (**29**) [40], isoangustone A (**30**) [41], and two additional compounds temporarily named compounds A (**31**), and B (**32**). Since several phenolics from licorice display potent antibacterial effects against methicillin-resistant *Staphylococcus aureus* (MRSA), and also show suppressing effects on the oxacillin resistance of MRSA, as we have reported previously [11], we have also investigated the effect of these licorice phenolics on VRE.

### 2.1. Structures of New Compounds

**Figure 3:**
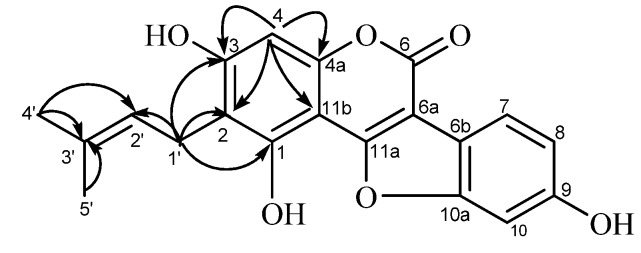
HMBC correlations observed for compound A (demethylglycyrol, **31**).

Compound A (**31**, Figure 3) was obtained as a pale-yellow microcrystalline powder. The high-resolution fast-atom bombardment mass spectroscopy (HR-FAB-MS) data (found *m*/*z* 353.0990 [M+H]^+^, calcd. 353.1025), indicated that this compound has a molecular formula C_20_H_16_O_6_. The UV spectrum showed absorption maxima at 259 (log ε, 4.45) and 345 nm (log ε, 4.23), which is similar to glycyrol, suggested that it has a 3-arylcoumarin or related coumestan skeleton as the chromophore. The ^1^H-NMR spectrum (Table 1) showed a one-proton singlet at *δ*_H_ 6.25 (H-4) and three protons forming an ABX system at *δ*_H_ 6.80 (1H, d, *J* = 2.4 Hz, H-10), *δ*_H_ 6.71 (1H, dd, *J* = 2.4, 8.4 Hz, H-8), and *δ*_H_ 7.25 (1H, d, *J* = 8.4 Hz, H-1) in the aromatic region, indicating the presence of penta-substituted and tri-substituted phenyl rings in the molecule. The absence of the H-11a signal (corresponding to H-4 of the 3-arylcoumarin skeleton) indicated the coumestan [29,31] structure for this compound. The remaining signals at *δ*_H_ 1.60, 1.77 (3H each, s, dimethyl at C-3'), *δ*_H_ 3.12 (2H, d, *J* = 6.6 Hz, methylene at C-1'), and *δ*_H_ 5.07 (1H, t, *J* = 6.6 Hz, methine at C-2') in the aliphatic proton region are corresponding to the presence of a *γ*,*γ*-dimethylallyl (prenyl) group in the molecule. The signal pattern in the ^1^H-NMR spectrum of **31** is similar to that of glycyrol (**15**) except that a methoxyl signal observed in the spectrum of **15** is absent in that of compound A. Therefore, the structure of demethylglycyrol (**31**) was assigned for this compound.

**Table 1:** 600 MHz NMR Spectroscopic Data for Demethylglycyrol (**31**) (acetone-d*_6_*, 27 °C), and 5,7-Di-*O*-methylluteone (**32**) (CD_3_OD, 27 °C).

	Demethylglycyrol (31)			5,7-Di-*O*-methylluteone (32)		
position	*δ*_C_, type	*δ*_H_ (*J* in Hz)	HMBC *^a^*	*δ*_C_, type	*δ*_H_ (*J* in Hz)	HMBC *^a^*
C-1	160.4, C					
C-2	113.9, C			170.1, CH	7.95, s	3, 4, 1'
C-3	158.5, C			119.9, C		
C-4	99.4, CH	6.25, s	2, 3, 4a, 11b	178.8, C		
C-4a	156.4, C			106.5, C		
C-5				159.2, C		
C-6	160.1, C			115.6, C		
C-6a	104.0, C					
C-6b	114.9, C					
C-7	98.4, CH	6.80, d (2.4)		160.4, C		
C-8	111.9, CH	6.71, dd (2.4, 8.4)		96.4, CH	6.34, s	6, 7, 4a, 8a
C-8a				156.7, C		
C-9	156.6, C					
C-10	120.0, CH	7.25, d (8.4)				
C-10a	156.1, C					
C-11a	158.6, C					
C-11b	104.1, C					
C-1'	23.2, CH_2_	3.12, d (6.6)	1, 2, 3, 2', 3'	117.5, C		
C-2'	125.1, CH	5.07, t (6.6)		160.0, C		
C-3'	130.5, C			128.2, CH	8.02, d (8.4)	2', 4'
C-4'	25.8, CH_3_	1.60, s	2',3', 5'	157.1, C		
C-5'	17.9, CH_3_	1.77, s	2', 3', 4'	116.8, CH	6.92, dd (2.4, 8.4)	1'
C-6'				103.3, CH	6.82, d (2.4)	2'
C-1''				23.5, CH_2_	3.38, d (6.6)	5, 6, 7, 2'', 3''
C-2''				125.2, CH	5.14, t (6.6)	
C-3''				130.9, C		
C-4''				17.7, CH_3_	1.73, s	2'', 3''
C-5''				25.7, CH_3_	1.64, s	2'', 3''
5-OCH_3_				61.3, CH_3_	3.78, s	5
7-OCH_3_				55.9, CH_3_	3.43, s	7

*^a^* HMBC correlations, optimized for 5 Hz, are from proton(s) stated to the optimized carbon.

The ^13^C-NMR spectrum showed five carbon signals ascribable to a prenyl group [*δ*_C_ 17.9, 25.8 (dimethyl at C-3'), *δ*_C_ 23.3 (C-1'), *δ*_C_ 125.1 (C-2'), *δ*_C_ 130.5 (C-3')], in addition to fifteen carbon signals assignable to the coumestan skeleton (see Table 1). Four carbon signals at *δ*_C_ 104.1 (C-11b), *δ*_C_ 113.9 (C-2), *δ*_C_ 156.4 (C-4a), and *δ*_C_ 158.5 (C-3) among the *sp*^2^ carbon signals are correlated with the aromatic proton at *δ*_H_ 6.25 (H-4) in the ^1^H-detected multiple bond correlation (HMBC) spectrum (Figure 3). On the other hand, correlations of the methylene proton signal at *δ*_H_ 3.12 (H-1') with the carbon signals at *δ*_C_ 113.9 (C-2), *δ*_C_ 158.5 (C-3), and *δ*_C_ 160.4 (C-1), along with the correlations with the allylic carbon signals at *δ*_C_ 125.1 (C-2') and *δ*_C_ 130.5 (C-3'), were also observed in the HMBC spectrum. These correlations are coincided with the location of the prenyl group at C-2.

The substitution pattern of the hydroxyl and prenyl groups on the coumestan skeleton was further confirmed by chemical evidence. Compound A (**31**) was methylated (see Experimental Section) to afford the methyl derivative **31a** (Figure 2), which was identical with the compound obtained by methylation of the known compound glycyrol (**15**). The structure of demethylglycyrol (**31)** for compound A was thus established.

**Figure 4:**
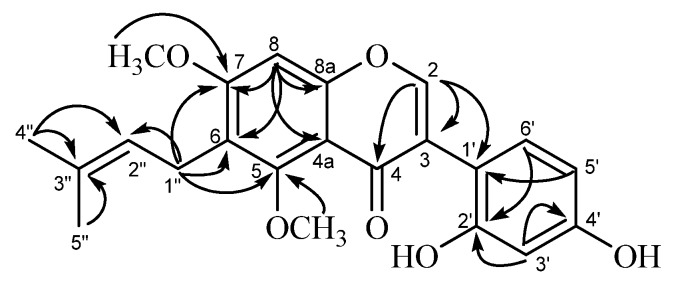
HMBC correlations observed for compound B (5,7-di-*O*-methylluteone, **32**).

Compound B (**32**, (Figure 4) was also obtained as a pale-yellow microcrystalline powder. Its HR-FAB-MS data (Found 383.1448 [M+H]^+^, Calcd 383.1495) indicated that this compound has a molecular formula C_22_H_22_O_6_. The UV spectrum of **32** with the absorption maxima at 258 (log ε, 4.08) 291sh, and 340 nm (log ε, 3.93) suggested that it has an isoflavone skeleton as the chromophore. The ^1^H-NMR spectrum showed two one-proton singlets a *δ*_H_ 7.97 (H-2) and *δ*_H_ 6.38 (H-8), and three protons forming an ABX system at *δ*_H_ 6.91 (1H, d, *J* = 2.4 H_Z_, H-3'), *δ*_H_ 6.99 (1H, dd, *J* = 2.4, 8.4 Hz, H-5'), and *δ*_H_ 8.02 (1H, d, *J* = 8.4 Hz, H-6') in the aromatic-proton region, corresponding to the isoflavone skeleton. The presence of the prenyl group was also indicated by a set of aliphatic protons at *δ*_H_ 1.60, 1.69 (3H each, s, dimethyl at C-3''), *δ*_H_ 3.21 (2H, d, *J* = 6.6 Hz, H-1''), and *δ*_H_ 5.14 (1H, t, *J* = 6.6 Hz, H-2''). The presence of the two methoxyl groups was shown by the signals at *δ*_H_ 3.40 and *δ*_H_ 3.76 (3H each, s). This ^1^H signal pattern for compound B was closely similar to that of licoricone (**28**), suggesting a structure isomeric to **32**. The ^13^C-NMR spectrum (in CD_3_OD) of compound (**32**), however, was discriminable from that of **28**. Compound B (**32**) showed the ^13^C signals of the isoflavone skeleton [*δ*_C_ 96.4 (C-8), *δ*_C_ 103.3 (C-3'), *δ*_C_ 106.5 (C-4a), *δ*_C_ 115.6 (C-6), *δ*_C_ 116.8 (C-5'), *δ*_C_ 117.5 (C-1'), *δ*_C_ 119.9 (C-3),*δ*_C_ 128.2 (C-6'),*δ*_C_ 156.7 (C-8a), *δ*_C_ 157.1 (C-4'), *δ*_C_ 159.2 (C-5), *δ*_C_ 160.0 (C-2'), *δ*_C_ 160.4 (C-7), *δ*_C_ 170.1 (C-2),*δ*_C_ 178.8 (C-4) (see Table 1)], in addition to the prenyl [*δ*_C_ 17.7, 25.4 (dimethyl at *δ*_C_ C-3")*δ*_C_ 23.5 (C-1"),*δ*_C_ 125.2 (C-2"), *δ*_C_ 130.9 (C-3")] and methoxyl groups (*δ*_C_ 55.9 and *δ*_C_ 61.3). Among the isoflavone skeleton signals of compound B (**32**), the chemical shift of C-2 (*δ*_C_ 170.1) showed a large difference from the corresponding carbon signal (*δ*_C_ 157.9) of licoricone (**28**) [39]. The assignment of C-2 in **32** was supported by the HMBC correlations of H-2 signal (*δ*_H_ 7.95) with carbon signals of C-3 (*δ*_C_ 119.9), C-4 (*δ*_C_ 178.8), and C-1' (*δ*_C_ 117.5), and correlations of H-5' signal (*δ*_H_ 6.92) with carbon signals of C-1' (*δ*_C_ 117.5). The HMBC spectrum also showed correlations of H-8 signal (*δ*_H_ 6.34) with carbon signals of C-4a (*δ*_C_ 106.5),C-6 (*δ*_C_ 115.6), C-8a (*δ*_C_ 156.7), and C-7 (*δ*_C_ 160.4), and that of H-1” (*δ*_H_ 3.38) with those of C-6 (*δ*_C_ 115.6), C-2” (*δ*_C_ 125.2),C-5 (*δ*_C_ 159.2), and C-7 (*δ*_C_ 160.4), substantiating the location C-6 for the prenyl group. The HMBC correlations (Figure 4) for the methoxyl groups [*δ*_H_ 3.78 with C-5 (*δ*_C_ 61.3), and *δ*_H_ 3.43 with C-7 (*δ*_C_ 55.9)] emphasized their locations at C-5 and C-7. The structure of 5,7-di-*O*-methylluteone (**33**) was thus assigned for compound B.

### 2.2. Antibacterial Effects of Licorice Phenolics on VRE

*E. faecium* FN-1 and *E. faecalis* NCTC 12201 strains were used in this study. The antibacterial effects of the licorice phenolics on the VRE strains were estimated using the liquid dilution method. Although except for linezolid almost all of the tested antibacterial standard drugs showed high minimum inhibitory concentration (MIC) values against at least one of the two used strains as shown in Table 2, among the licorice phenolics examined in this study, a 2-arylcoumarin, gancaonin I (**17**) showed potent antibacterial effects against *E. faecium* (MIC of 8 μg/mL), and *E. faecalis* (MIC of 16 μg/mL). Additionally, two 3-arylcoumarins, licoarylcoumarin (**9**) and glycycoumarin (**26**) showed low MIC values (16 μg/mL) for *E. faecium* and *E. faecalis*. An isoflavone semilicoisoflavone B (**14**), and an isoflavan glyasperin D (**29**) showed potent (16 μg/mL) to moderate MIC (32 μg/mL) for *E. faecium*. Flavonols, flavanones, and chalcones showed no or weak (~128 μg/mL) antibacterial effects. Noticeable that the compounds with two or three phenolic hydroxyl groups accompanied by a prenyl group showed potent anti-VRE effects relative to those of the other structural features. Since coumestans such as isoglycyrol (**18**) and demethlglycyrol (**31**) showed weak antibacterial effects relative to those of 3-arylcoumarins, the structural rigidity may cause decrease of the effects.

**Table 2:** Antibacterial effects of licorice phenolics on enterococci shown by their minimum inhibitory concentrations (μg/mL).

Compounds	Bacterial Strains	
	*Enterococcus faecium* FN-1	*Enterococcus faecalis* NCTC12201
Flavonols and related compounds		
Kaempferol-3- *O*-methyl ether (c)	>128	>128
Kaempferol ( **6**)	>128	>128
Isolicoflavonol ( **7**)	>128	128
Flavanones		
6"-O-Acetylliquiritin ( **11**)	>128	>128
Liquiritin ( **12**)	>128	128
Liquiritigenin ( **19**)	>128	>128
Chalcone		
Isoliquiritin ( **23**)	>128	>128
Isoflavones and Isoflavans		
Allolicoisoflavone B ( **2**)	>128	128
Formononetin ( **10**)	>128	>128
Semilicoisoflavone B ( **14**)	32	64
5,7-Di- *O*-Methylluteone (**32**)	64	128
Gancaonin G ( **20**)	64	128
6,8-Diprenylorobol ( **22**)	128	128
Glicoricone ( **25**)	>128	>128
Licoricone ( **28**)	128	>128
Glyasperin D ( **29**)	32	64
3-Arylcoumarins		
Isoglycycoumarin ( **8**)	64	>128
Licoarylcoumarin ( **9**)	16	16
Glycyrin ( **16**)	16	32
Glycycoumarin ( **26**)	16	16
Coumestans		
Glycyrol ( **15**)	>128	>128
Isoglycyrol ( **18**)	32	64
Demethlglycyrol ( **31**)	64	64
2-Arylcoumarones		
Gancaonin I ( **17**)	8	16
Licocoumarone ( **27**)	32	32
Others		
*p*-Hydroxybenzoic acid (**13**)	>128	128
Standard antibacterial drugs		
Erythromycin	>1024	>1024
Norfloxacin	>128	4
Vancomycin	>100	>100
Linezolid	2.5	2.5
Imipenem	>64	2
Tetracycline	64	128
Oxacillin	>1024	256
Gentamicin	>1024	>1024

## 3. Experimental

### 3.1. General

UV spectra were recorded on a JASCO V-530 spectrometer. ESI-MS measurements were performed on an API-4000 instrument. HR-FAB-MS measurements were conducted on a JEOL JMS-700 MStation with a mixture of *m*-nitrobenzyl alcohol and dithiothreitol as the matrix. ^1^H- and ^13^C-NMR spectra were recorded on an Agilent INOVA 600AS instrument (600 MHz for ^1^H, and 151 MHz for ^13^C), and the chemical shifts were given in *δ* (ppm) downfield from tetramethylsilane, based on those of the solvent signals [*δ*_H_ 2.04 and *δ*_C_ 29.8 for (CD_3_)_2_CO, and *δ*_H 3.30 and *δ*_C_ 49.8 for CD_3_OD). Optical rotations were measured on a JASCO DIP-1000 digital polarimeter. Normal-phase analytical HPLC was performed on an YMC SIL-003 (4.6 mm i.d. × 250 mm) column (YMC, Kyoto, Japan) with *n*-hexane–CH_3_OH–tetrahydrofuran–formic acid (55:33:11:1, *v*/*v*) containing oxalic acid (450 mg/L) as the eluent at the ambient temperature. Flow rate was set at 1.5 mL/min. Reversed-phase analytical HPLC was conducted on an YMC ODS-A 302 (4.6 mm i.d. × 250 mm) column with 10 mM H_3_PO_4_–10 mM KH_2_PO_4_–CH_3_CN–CH_3_COOH (35:35:28:2, *v*/*v*) as the eluent at 40 °C Flow rate was set at 1.0 mL/min. Preparative HPLC was performed on an YMC ODS-A324 (10 mm i.d. × 300 mm) column with H_2_O–CH_3_CN–CH_3_COOH (45:50:5, *v*/*v*) as the eluent. Detection for HPLC was effected with UV absorption at 280 nm. Silica gel (YMC), Toyopearl HW-40 (Coarse grade) (TOSOH, Tokyo, Japan), YMC-gel ODS-A (S, 75 μm) (YMC), and MCI-gel CHP-20P (Mitsubishi Chemical, Tokyo, Japan) were used for column chromatography. Sep-Pak C18 short cartridges (Waters, Milford, PA, USA) were also used for purification of compounds._

### 3.2. Plant Material

The crude drug used in this study is Tohoku Licorice (root and stolon of *Glycyrrhiza uralensis*), purchased from Tochimoto-tenkai-do (Osaka, Japan) (Lot No. 002009037), and the specimen GU-07112011(NEL) was kept at the Medicinal Plant Garden, Okayama University Graduate School of Medicine, Dentistry and Pharmaceutical Sciences.

### 3.3. Extraction and Isolation

Tohoku Licorice (1.0 kg) was pulverized and defatted with *n*-hexane (3 L × 2), and then with ethyl acetate (3 L × 2). A part (5 g) of the ethyl acetate extract (46.4 g) was subjected to the countercurrent distribution (*n* = 4, *r* = 4) with the solvent system CHCl_3_–CH_3_OH–H_2_O (7:13:8, *v*/*v*), to give 8 fractions [Lower phase (L1–L4) and upper phase (U1–U4) fractions with the following order of the polarity, L1 (3.93 g) < L2 (0.53 g) < L3 (101 mg) < L4 (69 mg) < U4 (50 mg) < U3 (59 mg) < U2 (71 mg) < U1 (51 mg)]. The L1 fraction was subjected to column chromatography on silica gel (3.0 cm i.d. × 30 cm) with increasing concentrations of CH_3_OH in CHCl_3_, to give 58 fractions. Combined fractions 10–13 (0.47 g) were subjected to column chromatography on ODS-gel (1.1 i.d. × 42 cm) with increasing concentrations of CH_3_OH in H_2_O, and the eluate with 20% CH_3_OH in H_2_O (155 mg) was further separated by column chromatography on MCI-gel CHP-20P (1.1 i.d. × 40 cm) with increasing concentrations of CH_3_OH in H_2_O, to give licoricidin (**1**) (10.2 mg), allolicoisoflavone B (**2**) (4.5 mg), and 3'-(*γ*,*γ*-dimethylallyl)-kievitone (**3**) (3.4 mg). The eluate with 25% CH_3_OH in H_2_O (43 mg) from the ODS-gel column was purified by preparative TLC on silica gel with CHCl_3_–CH_3_OH (9:1) to give isolicoflavonol (**7**) (3.1 mg), isoglycycoumarin (**8**) (2.5 mg), and licoarylcoumarin (**9**) (5.8 mg). The L2 fraction from the countercurrent distribution was subjected to column chromatography on silica gel (3.0 i.d. × 30 cm) with increasing concentrations of CH_3_OH in CHCl_3_, to give 25 fractions. Combined fractions 7–10 (0.18 g) were chromatographed on an ODS-gel column (1.1 i.d. × 42 cm) with increasing concentrations of CH_3_OH in H_2_O, and the eluate with 20% CH_3_OH in H_2_O (66 mg) was further chromatographed on an MCI-gel CHP-20P column (1.1 i.d. × 40 cm) with increasing concentrations of CH_3_OH in H_2_O, to give 7-*O*-methylluteone (**4**) (1.9 mg), kaempferol 3-*O*-methyl ether (**5**) (3.1 mg), and kaempferol (**6**) (6.8 mg). The eluate with 25% CH_3_OH in H_2_O (20 mg) from the ODS-gel column was purified by preparative TLC on silica gel with CHCl_3_–CH_3_OH (9:1), to give formononetin (**10**) (1.3 mg). The eluate with 30% CH_3_OH in H_2_O (10 mg) from the ODS-gel column was purified by preparative HPLC to give 6"-*O*-acetylliquiritin (**11**) (3.8 mg).

Separately, the ethyl acetate extract (40 g) from Tohoku Licorice was subjected to column chromatography on ODS-gel (2.2 i.d. × 75 cm) with increasing concentrations of CH_3_OH in H_2_O and then with increasing concentrations of CHCl_3_ in CH_3_OH. The eluate with 10% CHCl_3_ in CH_3_OH (4.1 g) was subjected to column chromatography on MCI-gel CHP-20P (2.2 i.d. × 45 cm) with increasing concentrations of CH_3_OH in H_2_O, and the eluate with 15% CH_3_OH in H_2_O (86 mg) was purified by preparative HPLC, to give liquiritin (**12**) (8.3 mg). The eluate with 30% CH_3_OH in H_2_O (53 mg) was purified by preparative TLC on silica gel with CHCl_3_–CH_3_OH, to give *p*-hydroxybenzoic acid (**13**) (6.9 mg) and semilicoisoflavone B (**14**) (2.0 mg). The eluate with 50% CHCl_3_ in CH_3_OH (3.6 g) was subjected to column chromatography on MCI-gel CHP-20P (2.2 i.d. × 45 cm) with increasing concentrations of CH_3_OH in H_2_O, and fractions 85 (48 mg), 94 (42 mg), 96 (40 mg), 137 (22 mg), 236–237 (19 mg), and 343–347 (59 mg) were respectively purified by preparative HPLC to give glycyrol (**15**) (4.1 mg), glycyrin (**16**) (15.4 mg) (from fraction 85), compound B (5,7-di-*O*-Methylluteone **32**) (3.5 mg), gancaonin I (**17**) (5.9 mg), isoglycyrol (**18**) (4.2 mg) (from fraction 94), liquiritigenin (**19**) (8.5 mg), gancaonin G (**20**) (3.1 mg), 3-(*p*-hydroxyphenyl)-7-methoxycoumarin (**21**) (1.2 mg), 6,8-diprenylorobol (**22**) (4.2 mg) (from fraction 96), isoliquiritin (**23**) (2.3 mg), 8-(*γ*,*γ*-dimethylallyl)-wighteone (**24**) (1.9 mg) (from fraction 137), compound A (Demethylglycyrol, **31**) (1.8 mg), glicoricone (**25**) (3.9 mg) (from combined fractions 236–237), glycycoumarin (**26**) (5.3 mg), licocoumarone (**27**) (7.9 mg), licoricone (**28**) (2.1 mg), glyasperin D (**29**) (3.5 mg) and isoangustone A (**30**) (4.5 mg) (from combined fractions 343–347).

### 3.4. Spectral Data

Compound A (demethylglycyrol, **31**): A pale-yellow, microcrystalline powder (MeOH); mp 265 °C; UV (MeOH) λ_max_ (log ε) 210 (4.47), 259 (4.45), 345 (4.23) nm; ^1^H- and ^13^C-NMR data see Table 1; ESI-MS *m*/*z* 353 ([M+H]^+^); HR-FAB-MS *m*/*z* 353.0990 ([M+H]^+^) (Calcd. for C_20_H_17_O_6_, 353.1025).

Compound B (5,7-di-*O*-methylluteone, **32**): A pale-yellow, microcrystalline solid (MeOH); mp 205 °C; UV (MeOH) λ_max_ (log ε) 210 (4.15), 258 (4.08), 291 (sh), 340 (3.93) nm; ^1^H- and ^13^C-NMR data see Table 1; ESI-MS *m*/*z* 383 ([M+H]^+^), HR-FAB-MS *m*/*z* 383.1448 ([M+H]^+^) (Calcd. for C_22_H_23_O_6_, 383.1495).

### 3.5. Methylation of Compound A and Glycyrol

Methylation of compound A (**31**) was carried out as shown in the literature [42]. Briefly, a solution of compound A (1.5 mg) in EtOH was treated by TMS-diazomethane at room temperature 3 h. The reaction mixture was concentrated under reduced pressure to a residue which was purified by TLC on silica gel (CHCl_3_–MeOH, 15:1, *v*/*v*), to give three compounds: glycyrol (0.5 mg), the monomethyl derivative of compound A (0.3 mg) (identified by ^1^H-NMR), and the corresponding trimethyl derivative (0.3 mg) (**31'**, Figure 2). Compound A-3Me (**31'**): ^1^H-NMR (acetone-*d*_6_): *δ*_H_ 1.66 and *δ*_H_ 1.81 (each 3H, s, -CH3 × 3), *δ*_H_ 3.44 (2H, d, *J* = 7 Hz, H-1"), *δ*_H_ 4.00, 4.01, 4.02 (each 3H, s, ‒OCH_3_ × 3), *δ*_H_ 5.22 (1H, t, *J* = 7 Hz, H-2''), *δ*_H_ 6.95 (1H, s, H-8), *δ*_H_ 7.05 (1H, dd, *J* = 2, 8 H_Z_, H-5'), *δ*_H_ 7.23 (1H, d,*J* = 2 Hz, H-3'), *δ*_H_ 7.82 (1H, d, *J* = 8 Hz, H-6'), *δ*_H_ 7.97 (1H, s, H-2). This compound is identical with that obtained by analogous treatment of glycyrol (**15**).

### 3.6. Antibacterial Assay

Estimation of antibacterial effects of licorice phenolics on vancomycin-resistant *Enterococcus* strains was carried out as has been described in the literature [9,43,44]. *Enterococcus faecium* FN-1 and *E. faecalis* NCTC 12201 used in this study were vancomycin resistant ones which were kindly provided by Dr. Y. Ike, Gunma University. The bacterial cells, pre-cultured in Mueller-Hinton broth at 37 °C under aerobic condition, were incubated in the presence of compounds with the concentrations obtained by serial two-fold dilution at 37 °C without shaking in 96-well plates in the same broth for 24 h. The inocula were adjusted to yield a final cell density of about 10^5^ CFU. The standard antibacterial drugs erythromycin, norfloxacin, vancomycin, linezolid, imipenem, tetracycline, oxacillin and gentamycin were used as reference compounds for the tested strains *Enterococcus faecium* FN-1 and *E. faecalis* NCTC 12201 in the present study. The minimum inhibitory concentrations (MICs) were estimated as the lowest concentrations where the bacterial cells were not observed visually. The MIC values were determined based on triplicate experiments.

## 4. Conclusions

Previous reports have shown that phenolics from licorice are potent antibacterial against MRSA [11,45], and some of them showed suppressing effects on the oxacillin resistance of MRSA [11]. To discover bioactive natural compounds from natural source, *Glycyrrhiza uralensis* was investigated, affording a new coumestan **31** and an isoflavone **32**, together with three known flavanols **5**‒**7**, three flavanones **11**, **12** and **19**, a chalcone **23**, eight isoflavones **2**, **10**, **14**, **20**, **22**, **25**, **28** and **32**, one isoflavan **29**, four 3-arylcoumarins **8**, **9**, **16** and **26**, three coumestans **15**, **18** and **31**, two 2-arylcoumarins **17** and **27** and *p*-hydroxybenzoic acid (**13**). Vancomycin-resistant *Enterococci* (VRE) is a serious drug-resistant bacteria, and just a few compounds such as linezolid, or a combination of quinupristin and dalfopristin have been used for treatments of diseases caused by them [12]. Therefore we have also investigated the effectiveness of the thirty two licorice phenolics isolated in this study on VRE, and we found that several compounds possesses moderate to potent antibacterial activity against VRE, and the 2-arylcoumarone gancaonin I (**17**) have the highest potency against the tested strains *E. faecium* (MIC of 8 μg/mL), and *E. faecalis* (MIC of 16 μg/mL), which is in agreement with the previously reported potent activity for a 2-arylcoumarin, gancaonin I (**17**) [15]. In addition to that, two 3-arylcoumarins, licoarylcoumarin (**9**) and glycycoumarin (**26**), also showed comparable antibacterial effects on *E*. *faecalis* (16 μg /mL). These findings could be useful in developing antibacterial agents from licorice and its various active phenolics. Besides the well-known traditional uses of licorice and the various reported biological effects [1,2,3,4,5,6,7,8], a recent study has added that several licorice phenolics exhibit higher tumor-specific cytotoxic effects [46]. However, further specific investigations on the safety of the pure licorice phenolics for human are awaited.

## References

[1] Isbrucker R.A., Burdock G.A. (2000). Risk and safety assessment on the consumption of licorice root (*Glycyrrhiza* sp.), its extract and powder as a food ingredient, with emphasis on the pharmacology and toxicology of glycyrrhizin. Regul. Toxicol. Pharm..

[2] Shen X.-P., Xiao P.-G., Liu C.-X. (2007). Research and application of *Radix Glycyrrhizae*. Asian J. Pharmacodyn. Pharmacokin..

[3] Asl M.N., Hosseinzadeh H. (2008). Review of pharmacological effects of *Glycyrrhiza* sp. and its bioactive compounds. Phytother. Res..

[4] Messier C., Epifano F., Genovese S., Grenier D. (2012). Licorice and its potential beneficial effects in common oro-dental diseases. Oral Dis..

[5] Villinski J.R., Bergeron C., Cannistra J.C., Gloer J.B., Coleman C.M., Ferreira D., Gafner S. (2014). Pyrano-isoflavans from *Glycyrrhiza uralensis* with antibacterial activity against *Streptococcus mutans* and *Porphyromonas gingivalis*. J. Nat. Prod..

[6] Gafner S., Bergeron C., Villinski J.R., Godejohann M., Kessler P., Cardellina J.H., Grenier D. (2011). Isoflavonoids and coumarins from *Glycyrrhiza uralensis*: Antibacterial activity against oral pathogens and conversion of isoflavans into isoflavan-quinones during purification. J. Nat. Prod..

[7] He J., Chen L., Heber D., Shi W., Lu Q.Y. (2006). Antibacterial Compounds from *Glycyrrhiza uralensis*. J. Nat. Prod..

[8] Fukai T., Marumo A., Kaitou K., Kanda T., Terada S., Nomura T. (2002). Anti- *Helicobacter pylori* flavonoids from licorice extract. Life Sci..

[9] Otsuka N., Liu M.-H., Shiota S., Ogawa W., Kuroda T., Hatano T., Tsuchiya T. (2008). Anti-methicillin resistant *Staphylococcus aureus* (MRSA) compounds isolated from *Laurus nobilis*. Biol. Pharm. Bull..

[10] Hatano T., Kusuda M., Inada K., Ogawa T., Shiota S., Tsuchiya T., Yoshida T. (2005). Effects of tannins and related polyphenols on methicillin-resistant *Staphylococcus aureus*. Phytochemistry.

[11] Hatano T., Shintani Y., Aga Y., Shiota S., Tsuchiya T., Yoshida T. (2000). Phenolic constituents of licorice. VIII. Structures of glicophenone and glicoiso-avanone, and effects of licorice phenolics on methicillin-resistant *Staphylococcus aureus*. Chem. Pharm. Bull..

[12] McNeil S.A., Clark N.M., Chandrasekar P.H., Kauffman C.A. (2000). Successful treatment of vancomycin-resistant *Enterococcus faecium* bacteremia with linezolid after failure of treatment with synercid (quinupristin/dalfopristin). Clin. Infect. Dis..

[13] Irani M., Sarmadi M., Bernard F. (2010). Leaves Antimicrobial activity of *Glycyrrhiza glabra* L. Iran J. Pharm. Res..

[14] Badr A.E., Omar N., Badria F.A. (2011). A laboratory evaluation of the antibacterial and cytotoxic effect of liquorice when used as root canal medicament. Int. Endod. J..

[15] Fukai T., Oku Y., Hano Y., Terada S. (2004). Antimicrobial activities of hydrophobic 2-Arylbenzofurans and an isoflavone against vancomycin-resistant *Enterococci* and methicillin-resistant *Staphylococcus aureus*. Planta Med..

[16] Park S.Y., Lim S.S., Kim J.K., Kang I.J., Kim J.S., Lee C., Park J.H.Y. (2010). Hexane-ethanol extract of *Glycyrrhiza uralensis* containing licoricidin inhibits the metastatic capacity of DU145 human prostate cancer cells. Br. J. Nutr..

[17] Tahara S., Shibaki S., Ingham J.L., Mizutani J. (1990). Further isoflavonoids from white lupin roots. Z. Naturforsch. C.

[18] O’Neill M.J., Adesanya S.A., Roberts M.F., Inez R.P. (1986). Inducible isoflavonoids from the lima bean, *Phaseolus lunatus*. Phytochemistry.

[19] Tahara S., Ingham J.L., Mizutani J. (1989). Metabolites of 7-O-methylluteone from *Botrytis cinerea*. Nippon Nogei Kagaku Kaishi.

[20] Valesi A.G., Rodriguez E., Vander Velde G., Mabry T.J. (1972). Methylated flavonols in *Larrea cuneifolia*. Phytochemistry.

[21] Xiao Z.P., Wu H.K., Wu T., Shi H., Hang B., Aisa H.A. (2006). Kaempferol and quercetin flavonoids from *Rosa rugosa*. Chem. Nat. Compd..

[22] Zheng Z.P., Cheng K.W., Chao J., Wu J., Wang M. (2008). Tyrosinase inhibitors from paper mulberry (*Broussonetia papyrifera*). Food Chem..

[23] Hatano T., Yasuhara T., Miyamoto T., Okuda T. (1988). Anti-human immunodeficiency virus phenolics from licorice. Chem. Pharm. Bull..

[24] Hatano T., Yasuhara T., Fukuda T., Noro T., Okuda T. (1989). Phenolic constituents of licorice. II. Structures of licopyranocoumarin, licoarylcoumarin and glisoflavone, and glisoflavone, and inhibitory effects of licorice phenolics on xanthine oxidase. Chem. Pharm. Bull..

[25] Chang Y.C., Nair M.G., Santell R.C. (1994). Microwave-mediated synthesis of anticarcinogenic isoflavones from soybeans. J. Agric. Food Chem..

[26] Shen F.J., Hu J.F., Yu Y.C., Xu Z.D. (1995). Studies on chemical constituents of *Glycyrrhiza uralensis* Fisch. Gaodeng Xuexiao Huaxue Xuebao.

[27] Nakanishi T., Inada A., Kambayashi K., Yoneda K. (1985). Flavonoid glycosides of the roots of *Glycyrrhiza uralensis*. Phytochemistry.

[28] Kiuchi F., Chen X., Tsuda Y. (1990). Four new phenolic constituents from licorice (root of *Glycyrrhiza* sp.). Heterocycles.

[29] Shul’ts E.E., Petrova T.N., Shakirov M.M., Chernyak E.I., Tolstikov G.A. (2000). Flavonoids of roots of *Glycyrrhiza uralensis* growing in Siberia. Chem. Nat. Compd..

[30] Nomura T., Fukai T., Wang Q.H. (1989). Four new prenylated flavonoids from aerial parts of *Glycyrrhiza uralensis*. Heterocycles.

[31] Shiozawa T., Urata S., Kinoshita T., Saitoh T. (1989). Revised structures of glycyrol and isoglycyrol, constituents of the root of *Glycyrrhiza uralensis*. Chem. Pharm. Bull..

[32] Fukai T., Wang Q.H., Kitagawa T., Litaka Y. (1989). Structures of six isoprenoids-substituted flavonoids, gancaonins F, G, R, I, glycyrol, and isoglycyrol from xibei licorice (*Glycyrrhiza* sp). Heterocycles.

[33] Krishnaswamy N.R., Seshadri T.R., Sharma B.R. (1966). Study of partial demethylation of some polymethoxy-3-phenylcoumarins and preparation of some new members. Indian J. Chem..

[34] Nkengfack A.E., Sanson D.R., Fomum Z.T., Tempesta M.S. (1989). 8-Prenylluteone, a prenylated isoflavone from *Erythrina eriotriocha*. Phytochemistry.

[35] Farag M.A., Porzel A., Wessjohann L.A. (2012). Comparative metabolite profiling and fingerprinting of medicinal licorice roots using a multiplex approach of GC–MS, LC–MS and 1D NMR techniques. Phytochemistry.

[36] Singhal A.K., Sharma R.P., Thyagarajan G., Herz W., Govindan S.V. (1980). New prenylated isoflavones and a prenylated dihydroflavonol from *Millettia pachycarpa*. Phytochemistry.

[37] Hatano T., Fukuda T., Miyase T., Noro T., Okuda T. (1991). Phenolic constituents of licorice. III. Structures of glicoricone and licofuranone, and inhibitory effects of licorice constituents on monoamine oxidase. Chem. Pharm. Bull..

[38] Zhu D.Y., Song G.Q., Jian F.X., Chang X.R., Guo W.B. (1984). Chemical constituents of *Glycyrrhiza uralensis* Fisch—Structures of isolicoflavonol and glycycoumarin. Huaxue Xuebao.

[39] Kaneta M., Saitoh T., Iitaka Y., Shibata S. (1984). Chemical studies on the oriental plant drugs. XXXVI. Structure of licoricone, a new isoflavone from licorice root. Chem. Pharm. Bull..

[40] Kwon H.J., Kim H.H., Ryu Y.B., Kim J.H., Jeong H.J., Lee S.W., Lee W.S. (2010). *In vitro* anti-rotavirus activity of polyphenol compounds isolated from the roots of *Glycyrrhiza uralensis*. Bioor. Med. Chem..

[41] Sil Lee Y., Ha Kim S., Kyu Kim J., Shin H.K., Kang Y.H., Park Y., Lim S.S. (2010). Rapid identification and preparative isolation of antioxidant components in licorice. J. Sep. Sci..

[42] Sasaki H., Kashiwada Y., Shibatav H., Takaishi Y. (2012). Prenylated flavonoids from the roots of Desmodium caudatum and evaluation of their antifungal activity. Planta Med..

[43] Nagoshi C., Shiota S., Kuroda T., Shiota S., Hatano T., Yoshida T., Kariyama R., Tsuchiya T. (2006). Synergistic effect of [10]-gingerol and aminoglycosides against vancomycin-resistant *Enterococci* (VRE). Biol. Pharm. Bull..

[44] Hossion A.M., Zamami Y., Kandahary R.K., Tsuchiya T., Ogawa W., Iwado A., Sasaki K. (2011). Quercetin diacylglycoside analogues showing dual inhibition of DNA gyrase and topoisomerase IV as novel antibacterial agents. J. Med. Chem..

[45] Fukai T., Marumo A., Kaitou K., Kanda T., Terada S., Nomura T. (2002). Antimicrobial activity of licorice flavonoids against methicillin-resistant Staphylococcus aureus. Fitoterapia.

[46] Ohno H., Araho D., Uesawa Y., Kagaya H., Ishihara M., Sakagami H., Yamamoto M. (2013). Evaluation of cytotoxiciy and tumor-specificity of licorice flavonoids based on chemical structure. Anticancer Res..

